# Association of Neisseria gonorrhoeae Plasmids With Distinct Lineages and The Economic Status of Their Country of Origin

**DOI:** 10.1093/infdis/jiaa003

**Published:** 2020-03-12

**Authors:** Ana Cehovin, Keith A Jolley, Martin C J Maiden, Odile B Harrison, Christoph M Tang

**Affiliations:** 1 Sir William Dunn School of Pathology, University of Oxford, Oxford, United Kingdom; 2 Department of Zoology, University of Oxford, Oxford, United Kingdom

**Keywords:** *Neisseria gonorrhoeae*, antimicrobial resistance, whole-genome sequencing, plasmids

## Abstract

Plasmids are vehicles for horizontal gene transfer between bacteria, and in *Neisseria gonorrhoeae* plasmids can mediate high-level antimicrobial resistance (AMR). Using genomic and phylogenetic analyses, we show that plasmids are widespread in a collection of 3724 gonococcal isolates from 56 countries, and characterized the conjugative, *β*-lactamase and cryptic plasmids. We found that variants of the conjugative plasmid (which can mediate tetracycline resistance) and the *β*-lactamase plasmid expressing TEM-135 are associated with distinct gonococcal lineages. Furthermore, AMR plasmids are significantly more prevalent in gonococci from less wealthy countries, highlighting the need for further studies. More than 94% of gonococci possess the cryptic plasmid, with its absence correlated with the presence of a novel chromosomal type IV secretion system. Our results reveal the extent of plasmid-mediated AMR in the gonococcus, particularly in less wealthy countries, where diagnostic and therapeutic options can be limited, and highlight the risk of their global spread.

## Teaser

Resistance plasmids are widespread in *Neisseria gonorrhoeae* in less wealthy countries. The presence and type of plasmid are closely associated with the core genome. Our results highlight how the cooperation between plasmids has important implications for the emergence of resistance.

**(See the Editorial Commentary by Kahler on pages 1762–3 and the Major Article by Harrison et al on pages 1816–25.)**

Plasmids are important vehicles for horizontal gene transfer (HGT) in bacteria, and frequently harbor genes encoding virulence factors, antimicrobial resistance (AMR), and properties that allow bacteria to survive in diverse niches [1]. Plasmids spread through bacterial populations by transformation and conjugation, resulting in the rapid dissemination of traits. Characterization of plasmids, including understanding their distribution in bacterial populations, is therefore key to understanding bacterial evolution, and in particular the spread of AMR.

*Neisseria gonorrhoeae* has developed resistance to all classes of antimicrobials [2]. This bacterium, the causative agent of the sexually transmitted infection gonorrhea, is a major global public health concern and a World Health Organization (WHO) priority antibiotic-resistant pathogen [3]. Complications from gonococcal disease include infertility, pelvic inflammatory disease, ectopic pregnancy, and neonatal conjunctivitis [2]. Gonococcal infection is also a cofactor for the acquisition and transmission of HIV [2].

Gonococcal AMR determinants are either chromosomally or plasmid-encoded [2], with recent research largely focusing on mechanisms of chromosomally mediated AMR, such as mosaic *penA* alleles [4]. However, *N. gonorrhoeae* can harbor 2 plasmids, the conjugative and *β*-lactamase, which can confer high-level resistance against tetracycline and *β*-lactams, respectively. The 2 AMR plasmids were first reported in gonococci in 1970s and 1980s [5], respectively, and had profound effects on the treatment of gonorrhea, resulting in a decreased use of benzyl penicillin and tetracycline. Although treatment failures with penicillin were often due to chromosomal mutations, tetracycline was discontinued as a first-line treatment in late 1980s owing to plasmid-mediated resistance [2]. In addition, the cryptic plasmid is highly prevalent in the gonococcus, even though it has no known function [5].

We propose a nomenclature for gonococcal plasmids, with the prefix *p* followed by an abbreviation for each plasmid (ie, pConj for the conjugative, p*bla* for the *β*-lactamase, and pCryp for the cryptic plasmid). Based on the presence or absence of *tetM* and restriction digest patterns, 3 types of pConj have been identified to date: (1) markerless (lacking *tetM*), (2) Dutch (*tetM* allele 1), and (3) American (*tetM* allele 2) [6]. The p*bla* and pCryp are small (<10 kb) and nonconjugative; however, p*bla* can be mobilized by pConj [7]. Several p*bla* have been isolated, all of which carry the TEM *β*-lactamase (*bla*_TEM_) and are derived from the prototypical Asia plasmid (7.4 kb) [8–10]. pCryp (4.2 kb) is not associated with any phenotype, even though it is thought to be present in about 90%–95% of gonococcal isolates [5, 11].

While chromosomal AMR genes, such as mosaic *penA* alleles, can lead to resistance against cephalosporins [4], plasmid-mediated AMR continues to pose a significant threat. A particular concern is that the most prevalent *β*-lactamase allele in *N. gonorrhoeae*, TEM-1, requires only 2 amino acid substitutions to become an extended-spectrum *β*-lactamase (ESBL) [12], which would have profound effects on the management of gonorrhea. Moreover, the TEM-135 allele, which carries an M182T substitution, is widespread among gonococci [13] and requires only a single change to become an ESBL [12].

In the current study, we characterized plasmids in a global collection of >3500 gonococcal isolates, and we compared their distribution with the phylogeny of the N. gonorrhoeae core genome. Of note, we found that the presence and type of plasmid are closely associated with the core genome. Our results highlight how cooperation between plasmids can have important implications for the emergence of AMR, with pConj and p*bla* often found together. In addition, we demonstrate that gonococcal AMR plasmids are particularly prevalent in low- and middle-income countries (LMICs), with this previously unrecognized epidemic of plasmid-mediated AMR highlighting the need for further characterization of gonococcal plasmids worldwide. 

## METHODS

### Whole-Genome Sequence Data and Assembly

Whole-genome sequence (WGS) data were evaluated from 3724 *N. gonorrhoeae* isolates (https://pubmlst.org/neisseria) from 56 countries, including 2075 isolates from the United Kingdom [14, 15], 391 from a global data set [16], 242 from the United States [17, 18], 103 from Kenya [19], and 836 unpublished WGSs (Supplementary Table 1 and Supplementary Figure 1). Fastq sequences from the European Nucleotide Archive were assembled using Velvet and VelvetOptimiser [20]. The resulting contigs were deposited at pubMLST.org/neisseria with metadata [21]. Mixed or incorrectly speciated samples were found by means of ribosomal MLST [22] and excluded.

### Core Genome Annotation and Visualization

The gonococcal core genome consists of 1668 loci [23] and yielded the N. gonorrhoeae core genome multilocus sequence typing (MLST) scheme (Ng_cgMLST), version 1.0 [23, 24]. Deposited WGSs were analyzed with the BIGSdb genomics platform [25], which has “autotagger” and “autodefiner” functions that scan WGS against defined loci. Each different allele at every locus is given a unique integer. Distance matrices, based on pairwise allelic mismatches, were generated, and genetic relationships between gonococci visualized with a minimum spanning algorithm using Grapetree software [26].

Because MLST does not discriminate between gonococcal populations owing to frequent recombination [27], clustering was used to identify gonococcal core genome groups [23]. Briefly, each isolate was assigned a core genome sequence type (ST), grouping them based on a threshold of allelic differences (from <5 to <500) with ≥1 member of the same group. The gonococcal population structure was resolved into distinct, reproducible, and stable groups using a threshold of 400 differences to cluster isolates (Ng_cgc_400) [23].

### Plasmid Annotation and Analysis

Loci from pConj, p*bla*, pCryp, and other mobile genetic elements, such as the VirB type IV secretion system (T4SS), are defined in PubMLST [19, 28] (Supplementary Table 2). Manual scanning and curation were used to identify loci and alleles, and their absence validated by examining relevant regions using Artemis version 17.0.1 (Wellcome Sanger Institute) [29].

Gene-by-gene comparison of plasmid loci was undertaken using the BIGSdb Genome Comparator tool enabling assembly of allelic profiles based on all loci in each plasmid. Each profile was given a unique number (plasmid ST). Owing to extensive variation in pConj, a new clustering scheme, *N. gonorrhoeae* conjugative plasmid (Ng_cp), was derived using tools for core genome analysis. Ng_cp groups plasmids using a threshold of <3, <5, <10, or less, locus differences on pConj and were visualized using Grapetree software [26]. The distribution of p*bla* was assessed by analyzing the *bla*TEM allele, because this plasmid frequently contains truncated loci. Plasmid alignments were built using Easyfig software and basic local alignment search tool (BLAST) Ring Image Generator [30]. Long-read sequence data were available for isolates 36248, 39124, 39114, 39097, and 39089, [19], and WHO isolates L, G, and N [31] (GenBank accession nos. LT591902.1, LT591899.1, and LT591912.1, respectively).

### Statistical Analysis

Mann-Whitney and × ^2^ tests were performed using GraphPad Prism software, version 7.

## RESULTS

### Core Genome Phylogenetic Analysis and Plasmid Characterization

**Figure 1.**
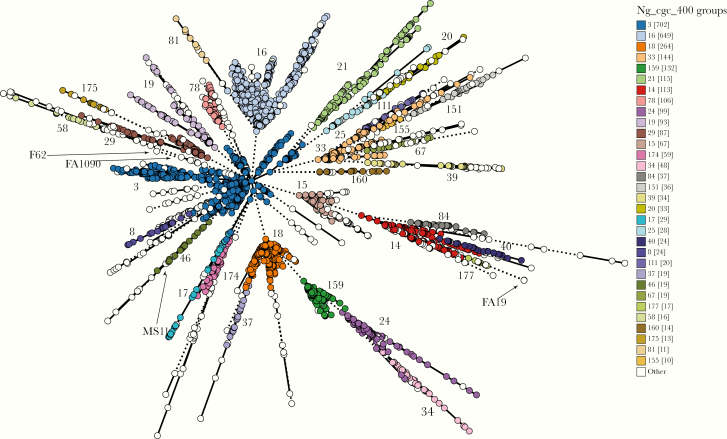
Population structure of *Neisseria gonorrhoeae,* with minimal spanning tree depicting Ng_cgMLST comparison of whole-genome sequence data of 3724 *N. gonorrhoeae* isolates used in this study. Each circle represents ≥1 isolate, and the size of the circle corresponds to the number of isolates. Only groups with ≥10 isolates are color coded and labeled, with the number of isolates in each group provided in brackets. The locations of common gonococcal laboratory strains are indicated.

Phylogenetic relationships among the 3724 gonococci in our collection were determined using the Ng_cgMLST scheme, which clusters isolates according to the alleles of 1668 core genome loci (Figure 1). Previously defined STs from MLST [32] associate with multiple isolate clusters [23], consistent with extensive recombination in *N. gonorrhoeae* [27]. Therefore, to identify distinct groups of gonococcal isolates, we used algorithms using different thresholds. Clusters containing isolates with ≤400 allelic differences (Ng_cgc_400) reproducibly resolve the gonococcal population into distinct, stable groups, which persist over time [23].

Next, we defined pConj, p*bla,* and pCryp through genes unique for each plasmid [6, 9, 33] (Table 1). pConj harbors genes encoding a T4SS (from NEIS2241 *traM* to NEIS2249 *traC*) [6] and a replicase *trfA* (NEIS2234). p*bla* contains the *β*-lactamase gene (*bla*TEM; NEIS2357) and replicase 1 (*rep1*; NEIS2358) [9], whereas pCryp encodes its replicase (*repA*; NEIS2952), replication initiation protein (NEIS2953), mobilization proteins (*mobB*, NEIS2958 and *mobC*, NEIS2951), relaxase, (*cppC* [33], NEIS2959), the *vapD/X* putative toxin-antitoxin system (TA; NEIS2956 and NEIS2955), and 2 hypothetical proteins (NEIS2954 and NEIS2957) (Table 1).

**Table 1.** Gonococcal Plasmid Types and their Variants

Plasmid	Defining Genes for Each Plasmid	Plasmid Variants	Defining Genes for Each Variant	Reference
pConj	NEIS2241 (*traM*) NEIS2242 (*traL*) NEIS2243 (*traK*) NEIS2244 (*traJ*) NEIS2245 (*traI*) NEIS2246 (*traG*) NEIS2247 (*traF*) NEIS2248 (*traE*) NEIS2249 (*traD*) NEIS2202 (*traC*) NEIS2234 (*trfA*)	pConj.1 (Ng_cp5: 2, 3, 5, 7, 10, 15, 19, 20)	*tetM* 2; NEIS2211; *trbK*; no NEIS2355	Current study
		pConj.2 (Ng_cp5: 11)	*tetM* 2; NEIS2211; no *trbK*; NEIS2355; *trbL* 45	Current study
		pConj.3 (Ng_cp5: 4, 17, 18, 22)	*tetM* 1; NEIS2211; NEIS2212; no NEIS2356	Current study
		pConj.4 (Ng_cp5: 13, 14, 8)	*tetM* 1; no NEIS2211; no NEIS2212; NEIS2356; *zeta_2* 43	Current study
		pConj.5 (Ng_cp5: 1, 23, 26, 27, 28, 30)	No *tetM*; no NEIS2211; no NEIS2212; NEIS2356; *zeta_2* 43; *trbI*_1; *trbF*_1/4	Current study
		pConj.6 (Ng_cp5: 6, 24, 25)	No *tetM*; no NEIS2211; no NEIS2212; NEIS2356; *zeta_2* 43; *trbL_8*; *trbI*_2; *trbF_2*	Current study
		pConj.7 (Ng_cp5: 16)	No *tetM*; no NEIS2211; no NEIS2212; NEIS2356; *zeta_2* 48	Current study
p*bla*	NEIS2357 (*bla*TEM) NEIS2358 (*rep1*)	p*bla*.As (Asia)	*mobA*; *mobC*; *rep2*	Dillon et al [34]
		p*bla*.Af (Africa)	*mobA*; *mobC*; No *rep2*	Pagotto et al [9]
		p*bla*.Ni (Nimes, France)	*mobA*; *mobC*; No *rep2*	Pagotto et al [9]
		p*bla*.NZ (New Zealand)	*mobA*; *mobC*; *rep2*	Pagotto et al [9]
		p*bla*.Rio (Rio de Janeiro, Brasil)	No *mobA* or *mobC*; *rep2*	Scharbaai-Vázquez et al [35]
		p*bla*.Jo (Johannesburg, South Africa)	*mobA*; No *mobC* or *rep2*	Müller et al [8]
		p*bla*.Au (Australia)	No *mobA* or *mobC*; *rep2* truncated	Trembizki et al [10]
pCryp	NEIS2951 (*mobC*) NEIS2952 (*repA*) NEIS2953 NEIS2954 NEIS2955 (*vapX*) NEIS2956 (*vapD*) NEIS2957 NEIS2958 (*mobB*) NEIS2959 (*cppC*)	None^a^	None^a^	…

Abbreviations: Ng_cp5, *Neisseria gonorrhoeae* conjugative plasmid 5.

^a^No variants identified; all pCryp have the same genetic content.

### Distribution of Plasmids Linked with Phylogeny of *N. gonorrhoeae*

**Figure 2.**
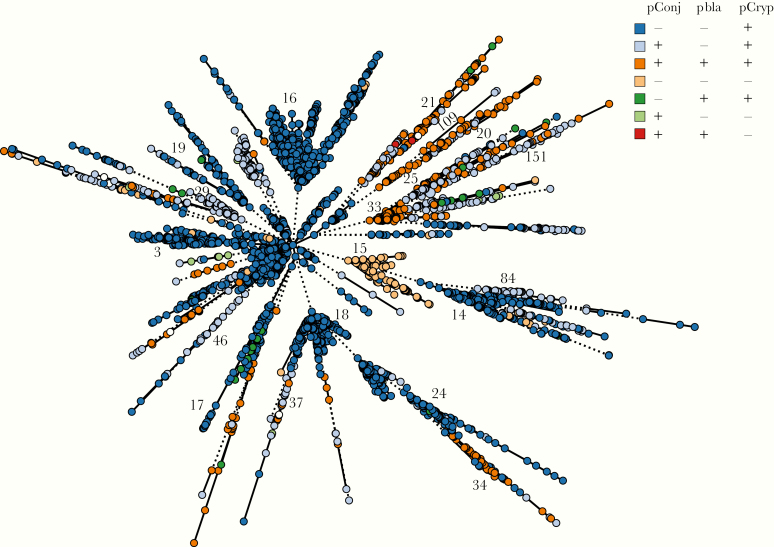
Plasmid distribution in *Neisseria gonorrhoeae*, with minimal spanning tree depicting Ng_cgMLST comparison of whole-genome sequence data. Each isolate is color coded according to the presence (+) or absence (-) of pConj, p*bla,* and pCryp. Ng_cgc_400 groups are indicated.

Only 10.7% of isolates (397 of 3724) carry all 3 plasmids, whereas about two-thirds of gonococci contain only pCryp (2440 of 3724 [65.5%]) (Supplementary Table 3 and Figure 2). A total of 17% of isolates (634 of 3724) harbor pCryp and pConj. Gonococci rarely contain pConj alone (14 of 3724 [0.4%]), whereas the combination of p*bla* and pConj (without pCryp) was found only twice (2 of 3724 [0.05%]). p*bla* is found more often with pConj (399 of 441 [90.5%]) than without (42 of 441 [9.5%]), with the association of p*bla* with pConj highly significant (*P* < .001; χ ^2^ test) (Figure 2 and Supplementary Table 3).

Of note, phylogenetic analyses show that plasmids are associated with distinct core genome groups (Ng_cgc_400 groups) (Figure 2). For example, groups 3, 14, 16, 18, 19, 24, and 17 harbor only pCryp, whereas pConj is prevalent in groups 29, 21, 46, 37, 34, 84, and related groups 33/151 and 20/25/109 (Figure 2). A total of 196 isolates (196 of 3724 [5.2%]), most of which belong to Ng_cgc_400 group 15 (69 of 196 [35%]), have no plasmid, whereas groups 3, 16 and 18, which are highly represented among UK and US isolates, do not harbor pConj (Figure 2 and Supplementary Figure 1).

### Identification of pConj Variants

pConj, which contains 50 loci, is the largest plasmid in *N. gonorrhoeae* (39–42 kb). A total of 1047 isolates (1047 of 3724 [28%]) harbor pConj, with almost half (503 of 1047 [48%]) containing *tetM,* which confers high-level resistance to tetracycline and doxycycline [19]. We identified 6 different *tetM* alleles (NEIS2210), with American (allele 2; 265 of 503 isolates [53%]), and Dutch (allele 1; 233 of 503 isolates [46%]) the most prevalent (Supplementary Table 4). Interestingly, these alleles cluster with particular Ng_cgc_400 groups and pConj plasmid variants (Supplementary Figure 2). Thus, the Dutch *tetM* allele is predominantly found in Ng_cgc_400 groups 29, 34 and 109, whereas the American *tetM* allele is associated with groups 21 and 151.

**Figure 3.**
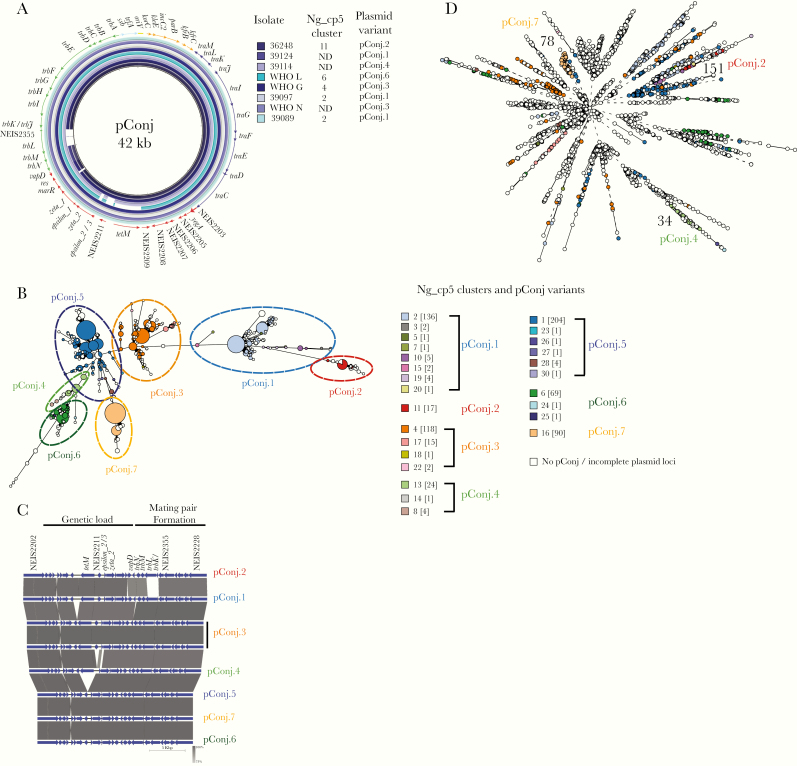
pConj variants in *Neisseria gonorrhoeae*. *A,* Alignment of long-read sequences of pConj from the World Health Organization (WHO) [30] and strains from coastal Kenya [19]. Each ring is color coded. Plasmid pEP5289 [6] was used as the reference sequence. Loci are colored as follows: green indicates mating pair formation; red, genetic load; blue, conjugative transfer; orange, inheritance control; and light blue, replication initiation [6]. Abbreviation: ND, not determined. *B,* Minimal spanning tree depicting comparison of pConj loci of all isolates used in this study. Each isolate on the phylogenetic tree is color coded according to the *Neisseria gonorrhoeae* conjugative plasmid 5 (Ng_cp5) cluster. pConj variants are indicated. Numbers in brackets represent the number of strains in each Ng_cp5 cluster. *C,* Alignment of genetic load and mating pair formation regions of pConj from strains 39113 (variant 2), 48828 (variant 1), 48349 and 27343 (variant 3), 36297 (variant 4), 31489 (variant 5), 31595 (variant 7), and 47574 (variant 6). Sequence identity between loci is depicted as shades in gray, as indicated. Missing or disparate loci are shown in white. *D,* Minimal spanning tree depicting Ng_cgMLST comparison of whole-genome sequence data of all isolates used in this study with Ng_cp5 groups labeled as in *B*. Plasmid variants and Ng_cgc_400 groups are indicated.

It has been thought that there are 3 forms of pConj based on the presence of Dutch or American *tetM*, or a markerless pConj [6]. However, alignment of pConj identified further differences (Figure 3A). To distinguish between different pConj, we implemented a plasmid sequence typing scheme. Unique sequences for every locus were assigned allele numbers, which were combined into allelic profiles and plasmid STs; this identified 215 plasmid STs for pConj (Supplementary Figure 3). Therefore, we clustered plasmids according to different thresholds of the number of allelic differences. Thus, the schemes Ng_cp3, Ng_cp5, and Ng_cp10 cluster plasmids on the basis of <3, <5, and <10 allelic differences and generated 37, 26, and 4 clusters of pConj, respectively (Figure 3B and Supplementary Figure 4). Ng_cp5 resolved differences between pConj (Figure 3B), and according to differences in genetic load and mating pair formation regions (Figure 3C), pConj was allocated into 7 variants, from pConj.1 to pConj.7.

pConj.1 and pConj.2 contain *tetM* allele 2 (American), pConj.3 and pConj.4 contain *tetM* allele 1 (Dutch), and pConj.5, pConj.6, and pConj.7 are markerless (Table 1). *trbK* is replaced with a hypothetical lipoprotein, NEIS2355 in pConj.2. pConj.4 contains *zeta_2 toxin* allele 43, and NEIS2212 is replaced with NEIS2356 (*epsilon_3 antitoxin*). pConj.5 and pConj.6 have *zeta_2 toxin* allele 43 but distinct alleles of *trbL*, *trbI,* and *trbF*. pConj.7 contains a truncated *zeta_2 toxin* (Table 1).

Interestingly, though pConj variants 1, 3, 5, and 6 are found across the gonococcal population, indicative of HGT, other pConj variants are associated with particular Ng_cgc groups. Thus, pConj.2, pConj.4, and pConj.7 are found exclusively in groups 151, 34, and 78, respectively (Figure 3D).

### Association of pbla with TEM-135 and Certain Core Genome Clusters

The classification of p*bla* is based on the presence or absence of mobilization and/or replication proteins (Table 1) [8–10, 13]. The different types of p*bla—*Asia (7.4 kb), Africa (5.6 kb), Nimes (6.8 kb), New Zealand (9.3 kb), Rio/Toronto (5.1 kb), Johannesburg (4.8 kb), and Australia (3.2 kb) [8–10, 13]*—*are thought to be derived from the 7.4-kb Asia plasmid (pJD4; accession no. U20374.1) [9]. We propose designating p*bla* using initials from their current names (eg, Asia plasmid, p*bla*.As) (Table 1). p*bla*.As includes genes encoding *β*-lactamase (NEIS2357; *bla*TEM), plasmid replication (NEIS2358; *repA* and NEIS2360; *repB*), mobilization (NEIS2961; *mobA* and NEIS2962; *mobC*), and several hypothetical proteins (NEIS2960, NEIS2963, and NEIS2964) (Supplementary Table 2).

A total of 11.8% isolates (441 of 3724) contain p*bla*. Repeated sequences within p*bla* prevented assembly of short-read sequences and use of plasmid sequence typing. Therefore, we typed p*bla* by analyzing *bla*TEM alleles. We identified 11 *bla*TEM alleles (Supplementary Table 5), of which allele 3, encoding TEM-1, is the most prevalent (202 of 441 of isolates with p*bla* [46%]), whereas 63 p*bla* (14.3%) harbor TEM-135 alleles, which carry a M182T substitution (alleles 2 and 8), with allele 11 containing a further substitution, A224T; the effect of this substitution is unknown [12]. In addition, 1 isolate has an E240K substitution (allele 14), which confers a minor change in resistance to *β*-lactams; combined with 2 further amino acid substitutions, this allele would encode an ESBL [12].

**Figure 4.**
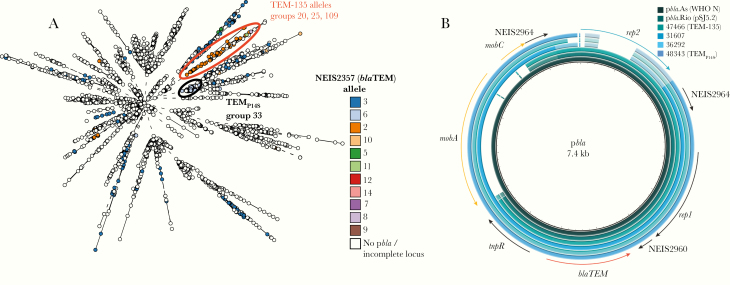
Distribution of *bla*TEM alleles and comparison of p*bla* plasmids in *Neisseria gonorrhoeae*. *A,* Distribution of *bla*TEM alleles on the minimal spanning tree depicting Ng_cgMLST comparison of whole-genome sequence data. The groups harboring TEM-135 and TEM_P14S_ are indicated. White circles indicate no p*bla* (n = 3292 isolates) or incompletely sequenced NEIS2357 locus (n = 81 isolates). *B,* Alignment of long-read (p*bla*.As and p*bla*.Rio) and short-read sequences of p*bla*. World Health Organization (WHO) N [31] (GenBank accession no. LT591911.1) and pSJ5.2 [35] (GenBank accession no. DQ355980.1) represent the Asia and Toronto plasmids, respectively. Each ring is color coded with WHO N used as the reference sequence. Arrows represent plasmid loci and are colored as follows: blue, *rep2*; red, *blaTEM*; orange, *mobA* and *mobC*; white blocks represent missing loci.

TEM-1 is widely distributed among gonococci containing p*bla*, whereas other TEM alleles are found in particular Ng_cgc_400 groups (Figure 4A). Thus, TEM-1 with P14S substitution (TEM_P14S_) is found in gonococci from the United Kingdom belonging to Ng_cgc_400 group 33 (62 of 144 [43%]), whereas TEM-135 is predominantly found within groups 20, 25, and 109 (43 of 71 [60%]) (Figure 4A). Furthermore, p*bla* containing TEM-135 have deletions in NEIS2961 (*mobA*) and NEIS2962 (*mobC*), which are also absent from p*bla*.Rio (Figure 4B) [13].

### A Novel T4SS in N. gonorrhoeae

**Figure 5.**
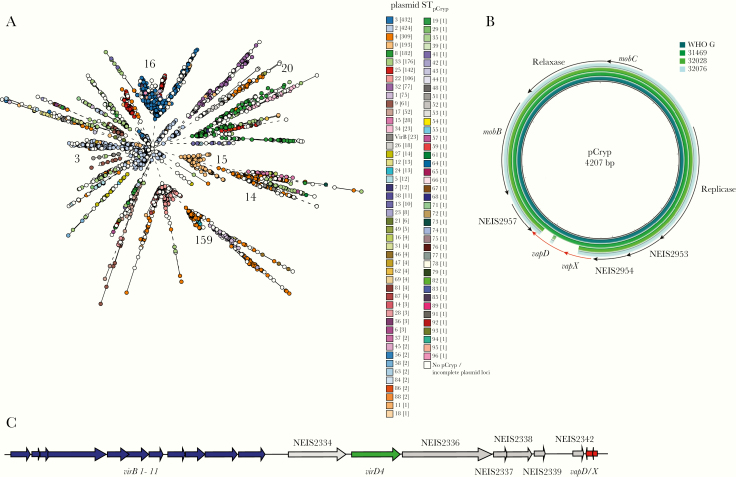
pCryp in *Neisseria gonorrhoeae*. *A,* Distribution of pCryp plasmid sequence types (ST_pCryp_) on the minimal spanning tree depicting Ng_cgMLST comparison of whole-genome sequence data. Ng_cgc_400 groups are indicated (the number of isolates in each group is shown in brackets). Isolates harboring the VirB type IV secretion system (T4SS) are displayed in dark gray. *B,* Alignment of pCryp with and without *vapD/X* loci. World Health Organization (WHO) G [31] and strain 31469 contain full-length pCryp, whereas strains 32028 and 32076, which harbor VirB T4SSs, have pCryp with disrupted *vapD/X*. *C,* Gene organization of VirB T4SS in *N. gonorrhoeae* depicting *vapD/X* in red.

Owing to low variation in pCryp, analysis of plasmid STs allows sufficient resolution to assess the spread of this plasmid (Figure 5A). In total, we identified 95 pCryp plasmid STs (ST_pCryp_), with their distribution generally following the gonococcal population structure. For example, 65% of isolates belonging to Ng_cgc group 16 harbor ST_pCryp_3 (422 of 650 [65%]), whereas 60% of group 3 isolates contain ST_pCryp_2 (421 of 705 [60%]). Conversely, ST_pCryp_4 (n = 309) is found in group 14 but also in phylogenetically more distant groups, such as 159 and 20, consistent with HGT (Figure 5A).

A total of 5.2% of isolates (193 of 3724) do not contain pCryp. Many of them belong to Ng_cgc group 15 (69 of 193 [36%]), which lacks all 3 plasmids, with most of them (66 of 69 [95%]) isolated in the United Kingdom between 1989 and 2014 [14, 15]. Of note, strains 32028 and 32076, which harbor pCryp lack *vapDX*, a putative TA system (Figure 5B). In addition, 23 gonococci that lack pCryp contain *vapDX* on the chromosome, located within a putative VirB T4SS locus (Figure 5C). This is the third T4SS identified in gonococcus, in addition to the T4SSs on the gonococcal genetic island [36] and on pConj [6].

### Relationship between AMR Plasmids and LMICs

**Figure 6.**
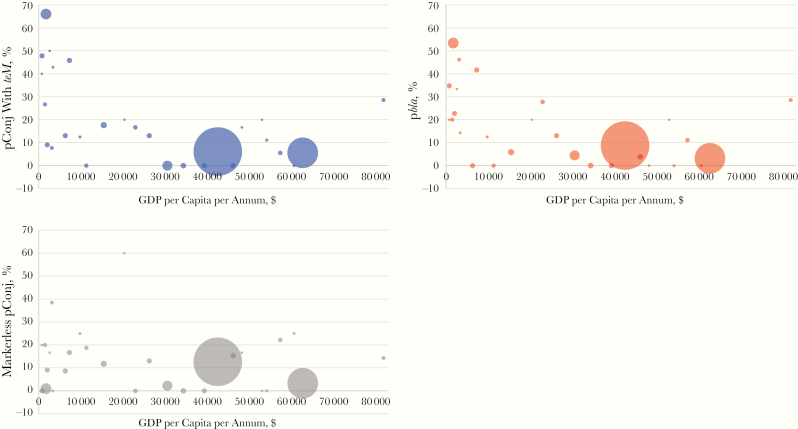
Global distribution of plasmids according to economic status of country of provenance. The percentages of pConj containing *tetM* (*blue circles*), p*bla* (*red circles*), and markerless pConj (*gray circles*) were calculated against the total number of *Neisseria gonorrhoeae* strains from each country (only countries with ≥5 strains were considered; the size of the circle corresponds to the total number of isolates in each country). Gross domestic product (GDP) data for fiscal year 2020 were obtained from the World Bank [37].

We also assessed the prevalence of plasmids in bacteria according to the economic status of country from where they had been isolated. Of note, pConj with *tetM* is significantly more frequent in isolates from LMICs compared with high-income countries (*P* = .007) (Figure 6 and Supplementary Table 6). For example, 30.3% of isolates from LMICs contain pConj with *tetM*, whereas in high-income countries, 6.1% of isolates have pConj with *tetM* (Supplementary Table 6). Conversely, the frequency of pConj without *tetM* has no relationship with a country’s gross domestic product (GDP) (*P* = .46) (Figure 6 and Supplementary Table 6). Therefore, the presence of genes conferring AMR is necessary for a plasmid to be associated with GDP.

## DISCUSSION

Gonorrhea is a major public health problem because of its impact on reproductive health, increasing AMR and the potential for untreatable gonorrhea in the near future [2]. Of particular concern is reduced susceptibility to third-generation cephalosporins, which are the mainstay of treatment [2], with recent research focused on chromosomally mediated AMR [2, 4]. In the current study, we characterized plasmids in *N. gonorrhoeae* from across the world and assessed their distribution in relation to gonococcal lineages and country of origin.

Plasmids play a vital role in disseminating AMR and often encode complex mechanisms to promote their stable inheritance and spread [1]. We provide the first global genomic analysis of plasmids in gonococci. Although many strains were obtained from the United Kingdom and the United States, our collection includes strains from >50 countries. There is a remarkably high prevalence of AMR plasmids in gonococci isolated in LMICs. This indicates that there is extensive spread and maintenance of plasmid-mediated AMR in gonococci circulating in LMICs and highlights the need for further studies to analyze *N. gonorrhoeae,* particularly from countries in South America, Africa, and Asia.

We analyzed WGS data deposited in the pubmlst.org/neisseria [25], which allows annotation of core and accessory genomes across the *Neisseria* genus. We were able to assess the diversity of plasmid-encoded genes and the association of certain plasmids with the gonococcal population structure. Our results demonstrate that distinct gonococcal core genomes are closely associated with particular plasmids. For example, certain types of pConj (variants 2, 4, and 7), TEM-135 and TEM_P14S_, and pCryp are found in particular Ng_cgc groups. Associations between plasmids and bacterial lineages have been described in other pathogens [38, 39]. Potential reasons for this include the presence of compensatory mutations, which mitigate the fitness costs of possessing a plasmid, chromosomally encoded restriction systems or Clustered Regularly Interspaced Short Palindromic Repeats (CRISPR)-Cas, and ancestral plasmid acquisition followed by clonal expansion; further studies are underway to examine these mechanisms.

We also demonstrate an association between Ng_cgc groups and the absence of plasmids. For example, isolates belonging to Ng_cgc_400 group 15 lack plasmids. In the absence of pCryp, isolates can harbor chromosomally encoded *vapD/X* in a novel VirB T4SS locus. T4SSs export DNA or proteins and can contribute to virulence in human pathogens such as *Helicobacter pylori* and *Bordetella pertussis* [40]. *vapD/X* is a putative TA system [41] usually present on pCryp. Gonococci can possess 2 *vapD* genes, 1 on pConj and the other associated with pCryp. The genes are 30% identical, and have homology to the CRISPR/Cas-associated protein Cas2, which forms a complex with Cas1 during the acquisition of foreign DNA into CRISPR loci that provide immunity against invading mobile genetic elements [42]. In addition, VapD homologues in other bacteria have endoribonuclease activity [41]. The presence of *vapD* on either pCryp or the VirB T4SS could inhibit the maintenance of other *vapD*-containing mobile genetic elements in the bacterium.

We identified 7 pConj variants, which differ in the genetic load and mating pair formation regions in genes encoding zeta/epsilon TA, and *trbK* and *trbL*. Zeta toxins are part of TA systems and interfere with peptidoglycan synthesis [43]. Of the 3 forms of *zeta/epsilon* TA found on pConj, only the *zeta_1/epsilon_1* TA has been studied so far [43]. The presence of different *zeta toxins* might influence retention of pConj variants, and/or the fitness or virulence of gonococci.

Certain Ng_cgc groups are associated with specific TEM alleles. For example, p*bla* in Ng_cgc_400 groups 20, 25, and 109 have TEM-135 alleles with a M182T amino acid substitution, which could develop into an ESBL with 1 further mutation [12]. The prospect of gonococcal TEM developing into an ESBL is worrying as gonococci can harbor ESBL *bla*_TEM_ in a laboratory setting [44], and we found evidence of further substitutions in TEM-135 enzymes (eg, *bla*TEM allele 11). It is important to note that certain *bla*_TEM_ alleles in *N. gonorrhoeae* can also harbor mutations that slow down the hydrolysis of penicillins, as in Canada plasmid pJRD20 [45]. Future work will determine the effect of substitutions in *bla*_TEM_ identified here on susceptibility to *β*-lactams. Overall, p*bla* is much more likely to be found in strains with pConj. Mechanistically, this is likely to be a consequence of the ability of pConj to comobilize p*bla* [7, 35]. Strains with TEM-135 also harbor variant 3 or 5 pConj, although the significance of this particular association is not known.

The high frequency of plasmid-encoded AMR among strains from LMICs is a particular concern, given the speed at which plasmids can spread in bacterial populations [5]; the high proportion of plasmid-mediated resistance in some of these countries has been observed before [46–48]. The prevalence of plasmids in LMICs could be driven by ≥2 factors. First, the lack of association between GDP and pConj without *tetM* indicates that only plasmids conferring resistance are under positive selection in LMICs. In these countries, diagnostic facilities can be limited, and syndromic management of sexually transmitted infections is often used [19], with doxycycline being prescribed in line with WHO guidelines for urethritis, proctitis, and cervicitis [49]. Therefore, pConj harboring *tetM* is likely to be maintained and propagated through selective pressure imposed by use of doxycycline. Second, it is possible that fitness costs resulting from a plasmid are mitigated in strains circulating in LMICs. Regardless of the reason for the high prevalence of pConj, this plasmid can facilitate the cotransfer of p*bla* through bacterial populations [7], which in some countries carry TEM alleles that are a single change away from encoding an ESBL. Therefore, our findings emphasize the need to prioritize the development of rapid diagnostic measures and combination therapy to prevent the emergence of plasmid-mediated resistance to third-generation cephalosporins [2].

In addition to AMR, plasmids can encode virulence factors [1]. Therefore the occurrence of specific plasmid variants could affect host-pathogen interactions. Indeed, *vapD* from *Haemophilus influenzae* can promote intracellular survival [50]. Similarly, *vapD* homologues on pCryp or pConj might enhance gonococcal intracellular survival [51]. Therefore, the characterization of plasmids, together with an understanding their distribution and phylogenetic relationships, should not only enhance our ability to monitor and predict the emergence and spread of AMR but also aid our understanding of the mechanisms underlying gonococcal virulence.

## Supplementary Data

Supplementary materials are available at *The Journal of Infectious Diseases* online. Consisting of data provided by the authors to benefit the reader, the posted materials are not copyedited and are the sole responsibility of the authors, so questions or comments should be addressed to the corresponding author.

jiaa003_suppl_Supplementary_Figure_1

jiaa003_suppl_Supplementary_Figure_2

jiaa003_suppl_Supplementary_Figure_3

jiaa003_suppl_Supplementary_Figure_4

jiaa003_suppl_Supplementary_Tables

## Notes

***Acknowledgments.*** We are grateful to Emeline Lawaree, PhD and Katy Poncin, PhD for their critical reading of the manuscript.

***Financial support.*** This work was supported by Oxford Martin School and the Wellcome Trust (grants 102908/Z/13/Z and 214374/Z/18/Z, and 104992/Z/14/Z).

***Potential conflicts of interest.*** All authors: No reported conflicts. All authors have submitted the ICMJE Form for Disclosure of Potential Conflicts of Interest. Conflicts that the editors consider relevant to the content of the manuscript have been disclosed.
