# Supplementary figures and images for: Association of Neisseria gonorrhoeae Plasmids With Distinct Lineages and The Economic Status of Their Country of Origin
Source: J Infect Dis. 2020 Mar 12;222(11):1826–36. doi: 10.1093/infdis/jiaa003 (PMC7653084; doi:10.1093/infdis/jiaa003)

# Country

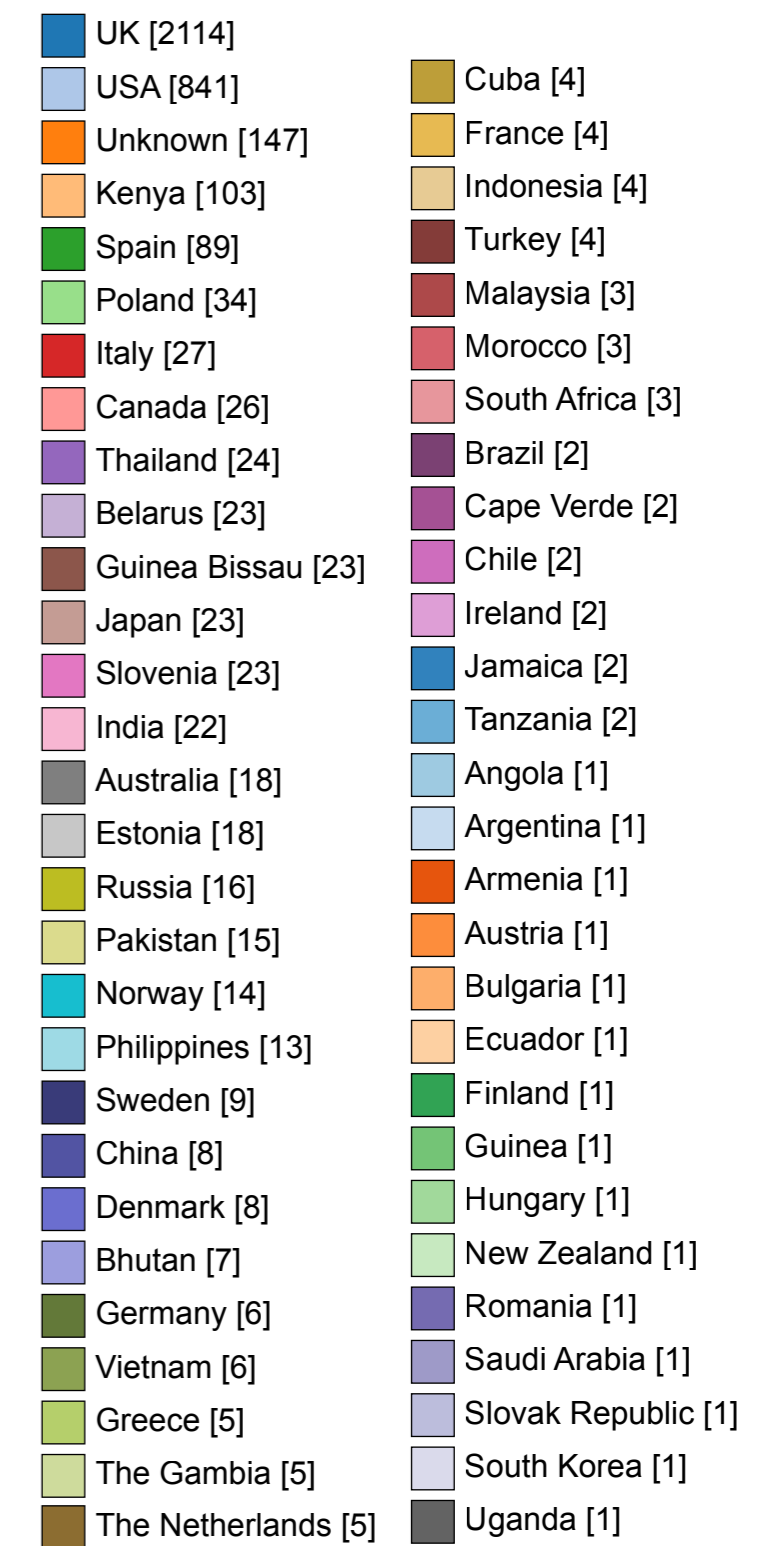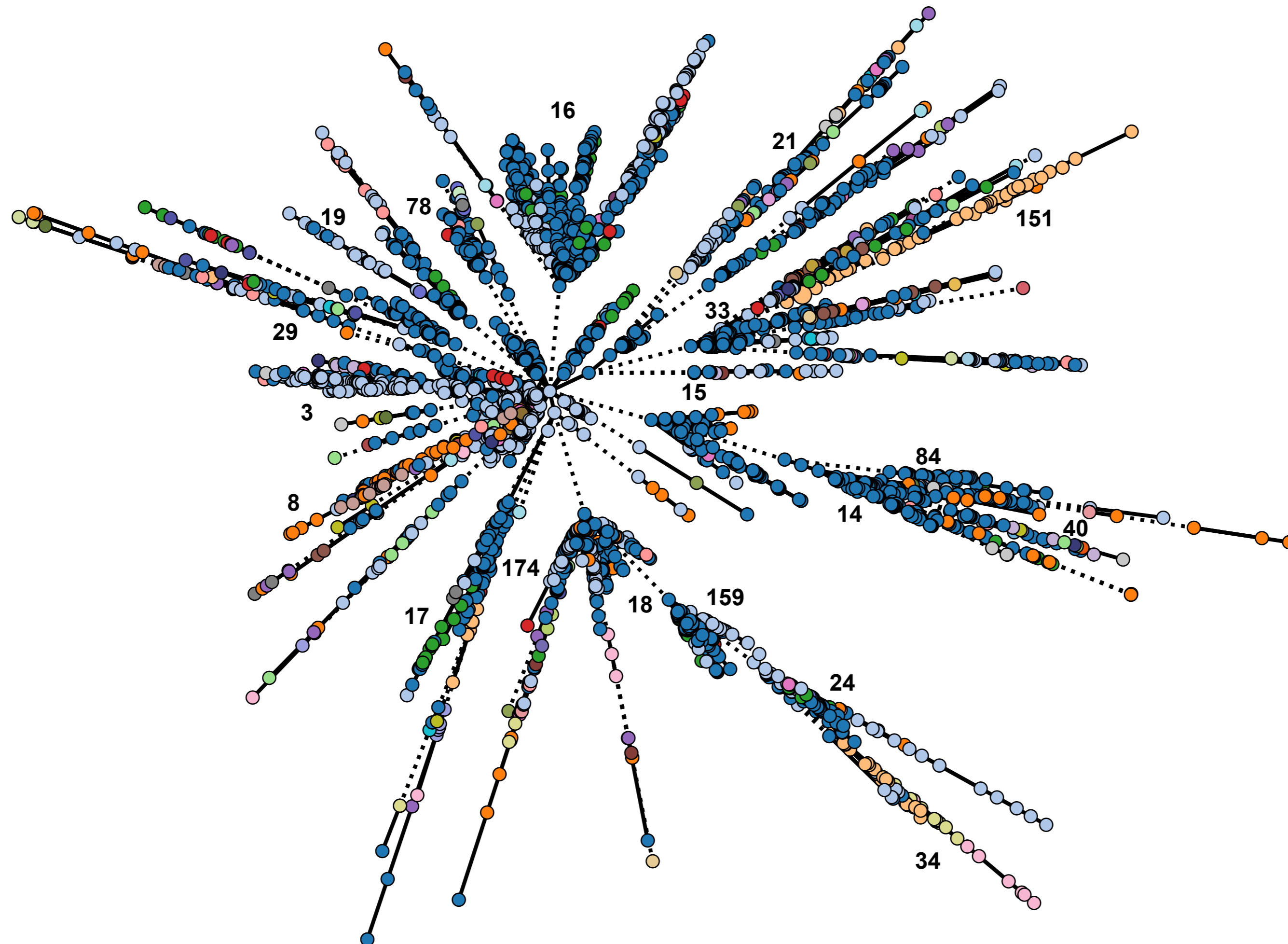

Supplement: jiaa003_suppl_Supplementary_Figure_1 [file jiaa003_suppl_supplementary_figure_1.pdf]

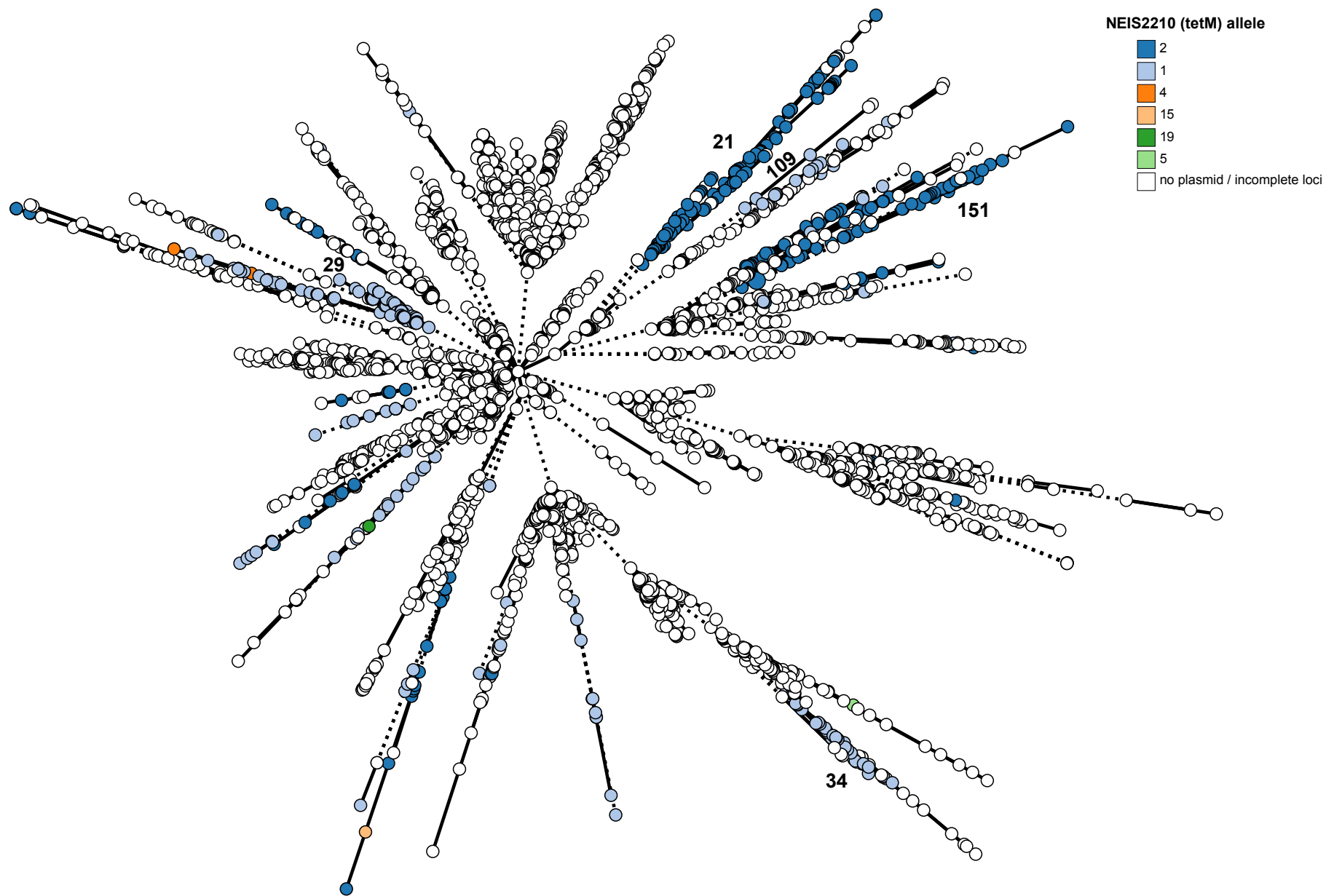

Supplement: jiaa003_suppl_Supplementary_Figure_2 [file jiaa003_suppl_supplementary_figure_2.pdf]

**A**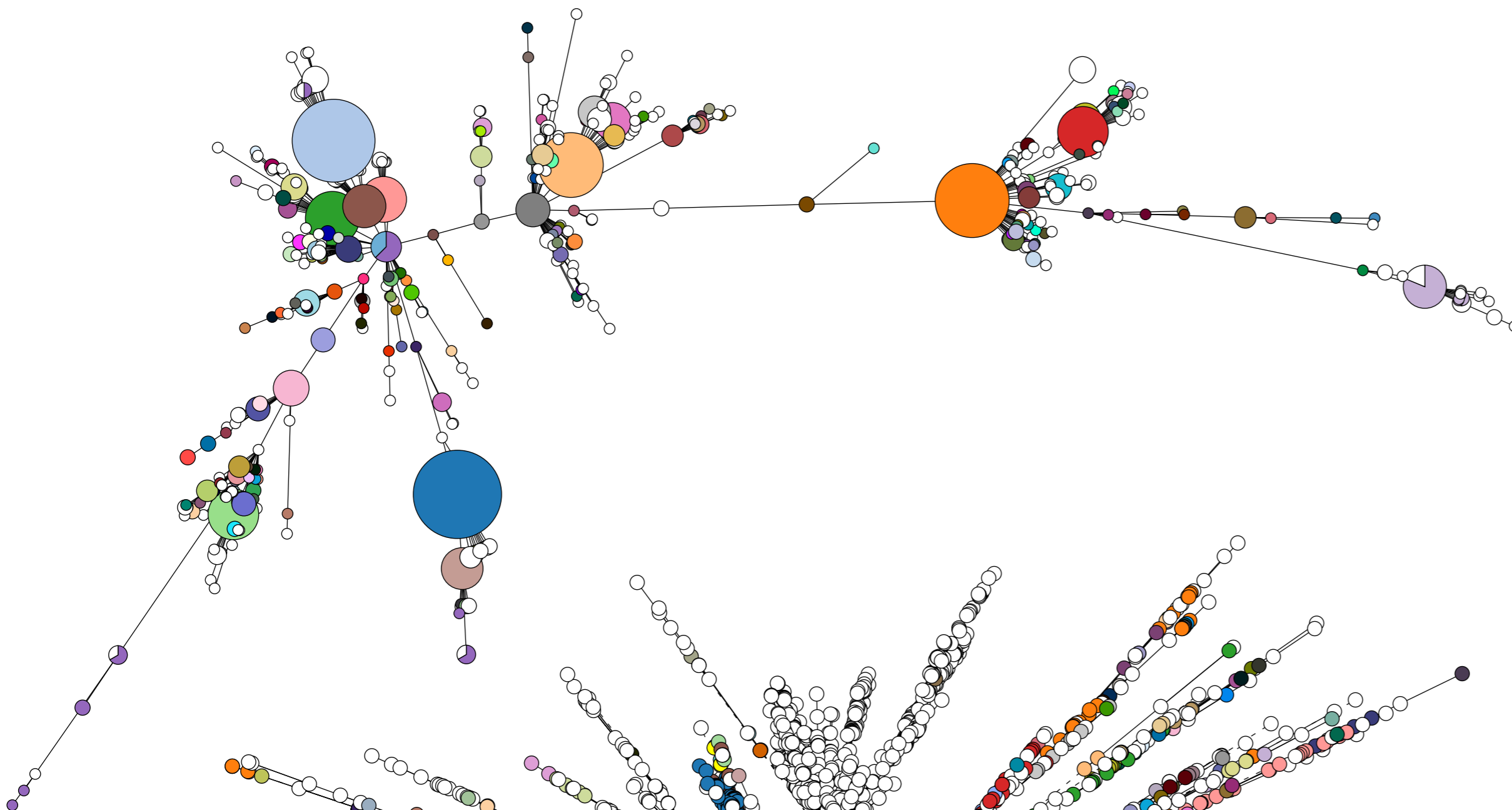**B**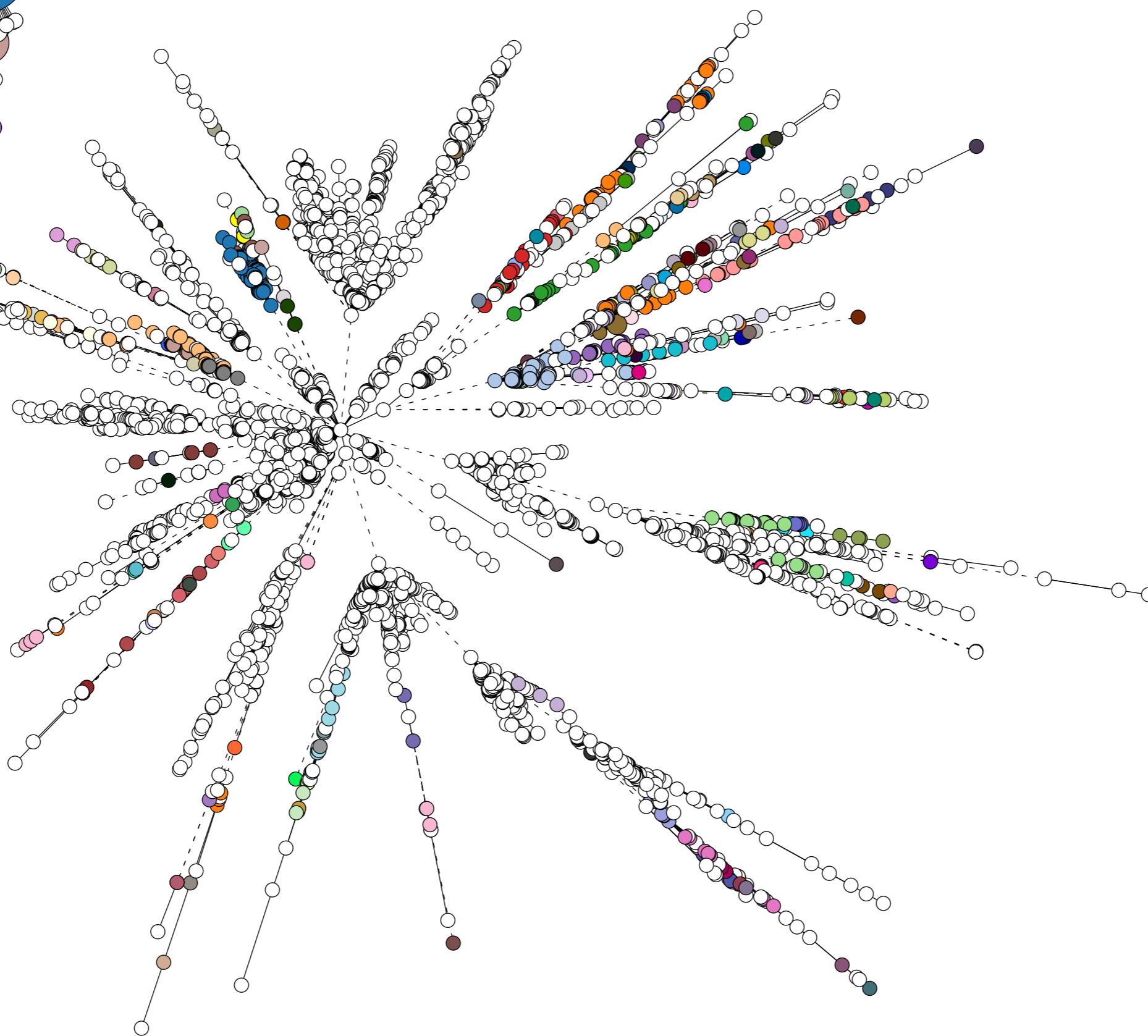**plasmid ST (pConj)**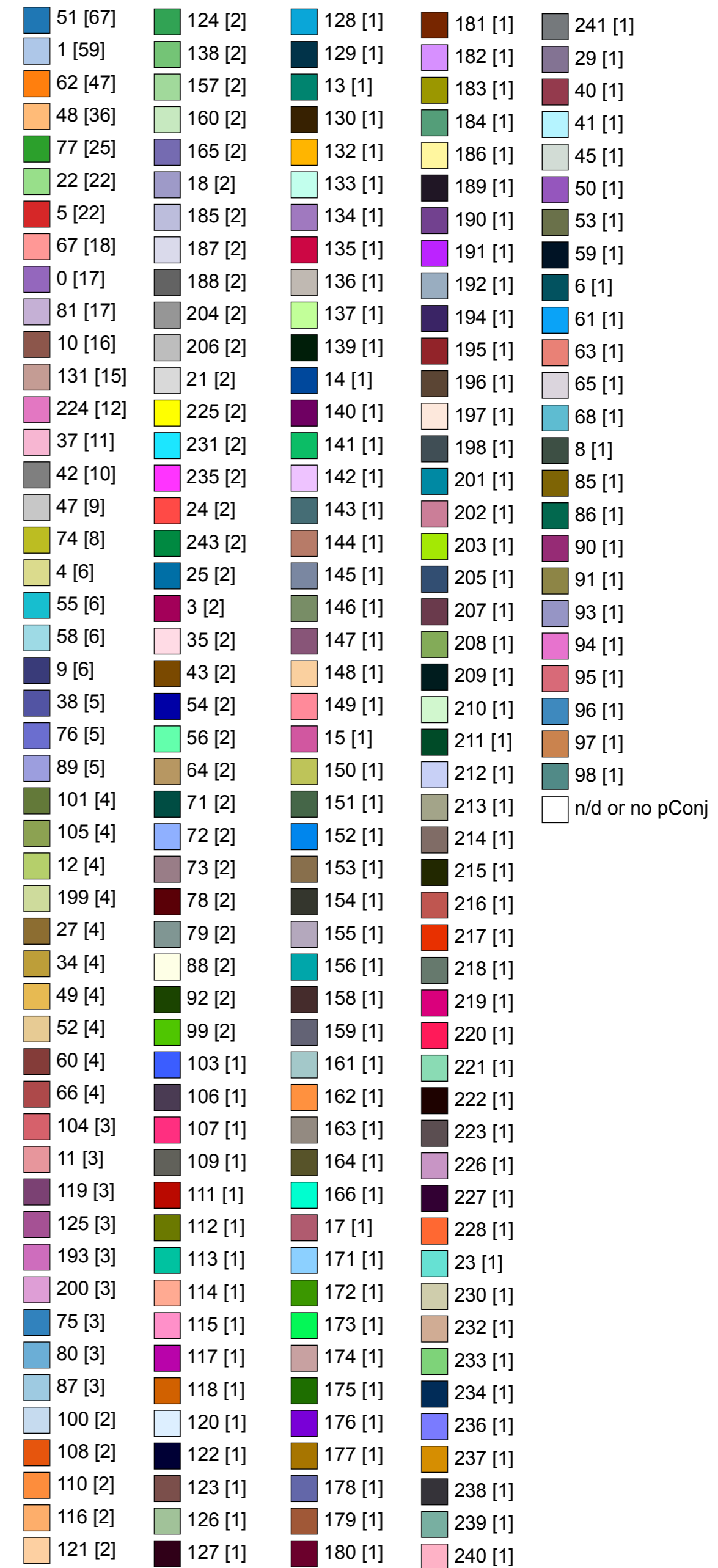

Supplement: jiaa003_suppl_Supplementary_Figure_3 [file jiaa003_suppl_supplementary_figure_3.pdf]

A

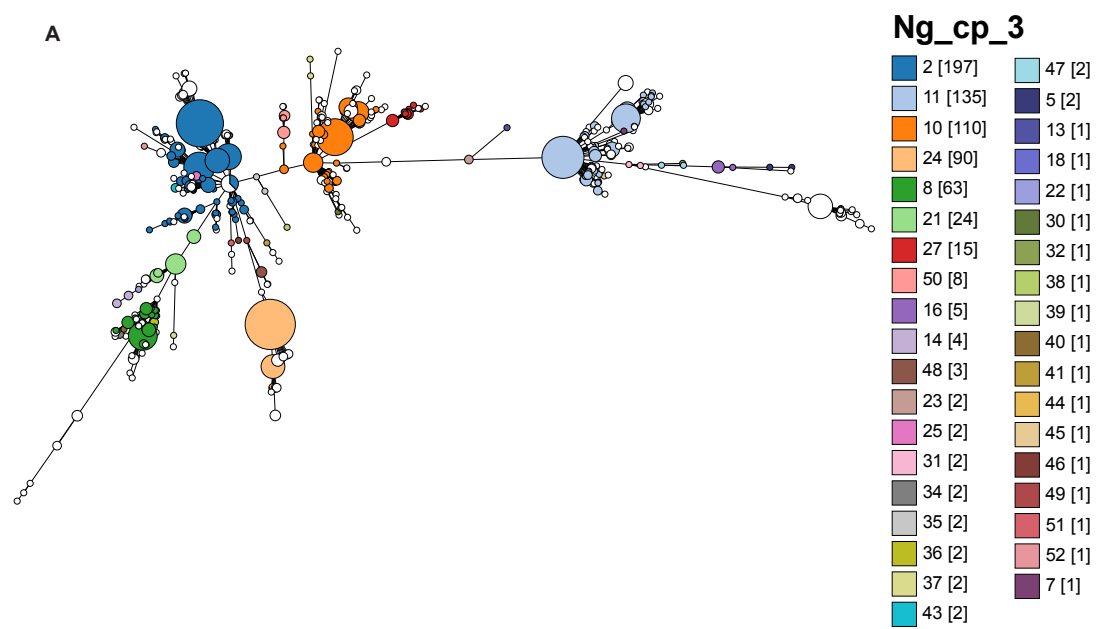

B

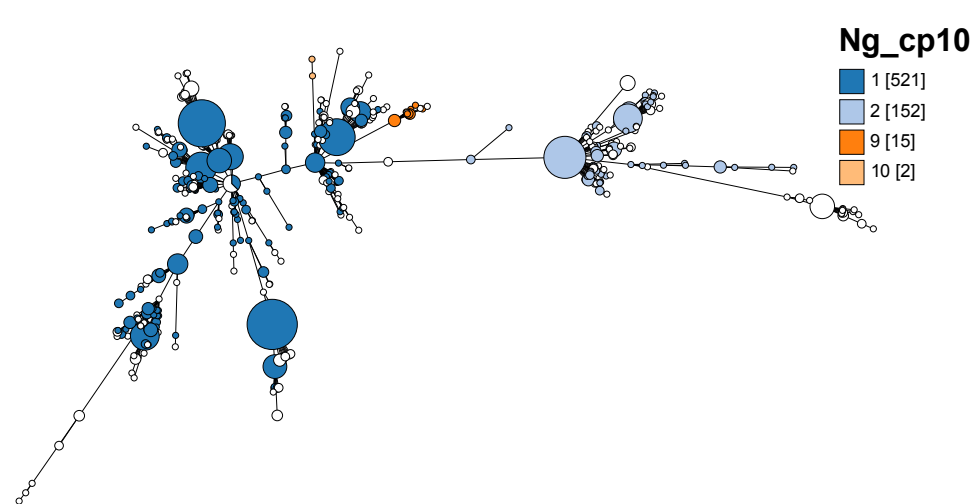

Supplement: jiaa003_suppl_Supplementary_Figure_4 [file jiaa003_suppl_supplementary_figure_4.pdf]
