# Supplementary material for: Association of Neisseria gonorrhoeae Plasmids With Distinct Lineages and The Economic Status of Their Country of Origin
Source: J Infect Dis. 2020 Mar 12;222(11):1826–36. doi: 10.1093/infdis/jiaa003 (PMC7653084; doi:10.1093/infdis/jiaa003)
Supplement: jiaa003_suppl_Supplementary_Tables [file jiaa003_suppl_supplementary_tables.docx]

**Supplementary Table 1.** Isolates used in this study.

Available to view or download from <https://figshare.com/s/a242f92b0aa96e614724>.

**Supplementary Table 2.** Plasmid and VirB T4SS loci annotation by BIGSdb.

| **Mobile Genetic Element** | **Locus** | **No. of alleles** | **Min length** | **Max length** | **Full name/product** |
| --- | --- | --- | --- | --- | --- |
| p*bla* | NEIS2357 | 21 | 861 | 864 | β-lactamase |
|  | NEIS2358 | 13 | 987 | 987 | replicase A |
|  | NEIS2359 | 9 | 530 | 531 | resolvase |
|  | NEIS2360 | 6 | 984 | 987 | replicase B |
|  | NEIS2960 | 6 | 168 | 168 | hypothetical protein |
|  | NEIS2961 | 15 | 1437 | 1438 | *mobA* |
|  | NEIS2962 | 5 | 297 | 298 | *mobC* |
|  | NEIS2963 | 10 | 258 | 258 | hypothetical protein |
|  | NEIS2964 | 2 | 258 | 258 | hypothetical protein |
| pConj | NEIS2202 | 103 | 2988 | 3396 | DNA primase |
|  | NEIS2203 | 48 | 495 | 495 | transcription elongation factor |
|  | NEIS2204 | 47 | 534 | 561 | *yegA* |
|  | NEIS2205 | 28 | 267 | 360 | hypothetical protein |
|  | NEIS2206 | 33 | 369 | 369 | hypothetical protein |
|  | NEIS2207 | 23 | 342 | 342 | hypothetical protein |
|  | NEIS2208 | 47 | 896 | 897 | putative DNA modification methylase |
|  | NEIS2209 | 27 | 474 | 477 | hypothetical protein |
|  | NEIS2210 | 19 | 1723 | 1935 | *tetM* |
|  | NEIS2211 | 9 | 522 | 522 | hypothetical protein |
|  | NEIS2212 | 4 | 255 | 255 | epsilon_2 antitoxin |
|  | NEIS2213 | 52 | 1209 | 1213 | zeta_2 toxin |
|  | NEIS2214 | 10 | 186 | 186 | hypothetical protein |
|  | NEIS2215 | 39 | 1206 | 1206 | zeta_1 toxin |
|  | NEIS2216 | 16 | 312 | 432 | *marR* |
|  | NEIS2217 | 22 | 555 | 555 | resolvase |
|  | NEIS2218 | 15 | 333 | 336 | *vapD* |
|  | NEIS2356 | 14 | 249 | 249 | epsilon_3 antitoxin |
|  | NEIS2219 | 36 | 600 | 600 | *trbN* |
|  | NEIS2220 | 36 | 570 | 573 | *trbM* |
|  | NEIS2221 | 61 | 1533 | 2193 | *trbL* |
|  | NEIS2222 | 4 | 141 | 141 | *trbK* |
|  | NEIS2223 | 36 | 762 | 777 | *trbJ* |
|  | NEIS2224 | 99 | 1338 | 1452 | *trbI* |
|  | NEIS2225 | 26 | 411 | 411 | *trbH* |
|  | NEIS2226 | 44 | 867 | 867 | *trbG* |
|  | NEIS2227 | 35 | 702 | 708 | *trbF* |
|  | NEIS2228 | 95 | 2565 | 2565 | *trbE* |
|  | NEIS2229 | 16 | 318 | 318 | *trbD* |
|  | NEIS2230 | 26 | 384 | 384 | *trbC* |
|  | NEIS2231 | 45 | 999 | 1020 | *trbB* |
|  | NEIS2232 | 18 | 366 | 366 | *trbA* |
|  | NEIS2355 | 7 | 231 | 231 | hypothetical protein |
|  | NEIS2233 | 27 | 383 | 390 | *ssb* |
|  | NEIS2234 | 42 | 879 | 882 | *trfA* |
|  | NEIS2235 | 16 | 348 | 348 | *korC* |
|  | NEIS2236 | 17 | 245 | 246 | *kleE* |
|  | NEIS2237 | 36 | 756 | 756 | *incC2* |
|  | NEIS2238 | 45 | 918 | 918 | *parB* |
|  | NEIS2239 | 20 | 357 | 357 | *kfrB* |
|  | NEIS2240 | 28 | 537 | 537 | *kfrC* |
|  | NEIS2241 | 23 | 480 | 483 | *traM* |
|  | NEIS2242 | 41 | 726 | 726 | *traL* |
|  | NEIS2243 | 29 | 201 | 483 | *traK* |
|  | NEIS2244 | 26 | 378 | 378 | *traJ* / *ori*T |
|  | NEIS2245 | 97 | 1107 | 2395 | *traI* |
|  | NEIS2246 | 67 | 1889 | 1891 | *traG* |
|  | NEIS2247 | 43 | 510 | 513 | *traF* |
|  | NEIS2248 | 92 | 2195 | 2196 | *traD* |
|  | NEIS2249 | 13 | 165 | 165 | *traC* |
| pCryp | NEIS2951 | 15 | 252 | 252 | *mobC* |
|  | NEIS2952 | 21 | 648 | 989 | replicase |
|  | NEIS2953 | 22 | 243 | 246 | HTH domain protein |
|  | NEIS2954 | 12 | 246 | 294 | hypothetical protein |
|  | NEIS2955 | 22 | 186 | 195 | *vapX* |
|  | NEIS2956 | 31 | 186 | 279 | *vapD* |
|  | NEIS2957 | 30 | 156 | 255 | hypothetical protein |
|  | NEIS2958 | 35 | 498 | 642 | *mobB* |
|  | NEIS2959 | 48 | 330 | 624 | relaxase |
|  | NEIS2323 | 18 | 804 | 819 | *virB1* |
|  | NEIS2324 | 8 | 330 | 330 | *virB2* |
|  | NEIS2325 | 11 | 429 | 429 | *virB3* |
|  | NEIS2326 | 37 | 2421 | 2430 | *virB4* |
|  | NEIS2327 | 14 | 684 | 684 | *virB5* |
|  | NEIS2328 | 15 | 999 | 1002 | *virB6* |
|  | NEIS2329 | 3 | 582 | 582 | *virB7* |
|  | NEIS2330 | 17 | 720 | 720 | *virB8* |
| VirB T4SS | NEIS2331 | 11 | 816 | 816 | *virB9* |
|  | NEIS2332 | 30 | 1296 | 1335 | *virB10* |
|  | NEIS2333 | 23 | 1098 | 1098 | *virB11* |
|  | NEIS2334 | 34 | 2379 | 2397 | *topA* |
|  | NEIS2335 | 28 | 2001 | 2013 | *virD4* |
|  | NEIS2336 | 43 | 2436 | 3711 | *traC* |
|  | NEIS2337 | 11 | 537 | 564 | hypothetical protein |
|  | NEIS2338 | 22 | 1041 | 1044 | relaxase |
|  | NEIS2339 | 13 | 453 | 495 | hypothetical protein |
|  | NEIS2340 | 70 | 420 | 420 | *stbB* |
|  | NEIS2341 | 42 | 237 | 237 | *stbC* |
|  | NEIS2342 | 11 | 450 | 453 | hypothetical protein |
|  | NEIS2343 | 28 | 780 | 780 | hypothetical protein |
|  | NEIS2344 | 6 | 663 | 663 | hypothetical protein |
|  | NEIS2345 | 32 | 360 | 360 | hypothetical protein |
|  | NEIS2347 | 1 | 192 | 192 | hypothetical protein |
|  | NEIS2348 | 7 | 402 | 447 | hypothetical protein |
|  | NEIS2349 | 8 | 618 | 618 | hypothetical protein |
|  | NEIS2350 | 7 | 288 | 552 | *trbM* |
|  | NEIS2351 | 9 | 744 | 747 | *traL* |
|  | NEIS2352 | 9 | 468 | 468 | hypothetical protein |
|  | NEIS2353 | 7 | 342 | 342 | hypothetical protein |
|  | NEIS2354 | 14 | 732 | 732 | integrase |

**Supplementary Table 3.** Prevalence of plasmid combinations in our dataset.

| **Plasmid combinations** | **No. of isolates** | **% of total** |
| --- | --- | --- |
| pCryp only | 2440 | 65.5 |
| pCryp and pConj | 634 | 17 |
| pCryp, pConj and p*bla* | 397 | 10.7 |
| No plasmid | 195 | 5.2 |
| p*bla* and pCryp | 42 | 1.1 |
| pConj only | 14 | 0.4 |
| p*bla* and pConj | 2 | 0.05 |

**Supplementary Table 4.** Prevalence of *tetM* alleles in our dataset.

| ***tetM* (NEIS2210) allele** | **Number of isolates** | **% of total** |
| --- | --- | --- |
| 2 (American) | 265 | 7.12 |
| 1 (Dutch) | 233 | 6.26 |
| 4 | 2 | 0.05 |
| 5 | 1 | 0.03 |
| 15 | 1 | 0.03 |
| 19 | 1 | 0.03 |

**Supplementary Table 5.** Prevalence of *bla*TEM alleles in our dataset.

| ***bla*TEM (NEIS2357) allele** | **TEM allele^1*^** | **Amino acid substitutions relative to TEM-1^*^** | **Number of isolates** | **% of total** |
| --- | --- | --- | --- | --- |
| 3 | TEM-1 | n/a | 202 | 5.42 |
| 6 | n/a | P12S | 68 | 1.83 |
| 2 | TEM-135 | M182T | 62 | 1.66 |
| 10 | n/a | Q5; H6Y | 14 | 0.38 |
| 5 | n/a | G228S | 2 | 0.05 |
| 7 | n/a | P14T | 1 | 0.03 |
| 8 | TEM-135 | M182T | 1 | 0.03 |
| 9 | TEM-1 | n/a | 1 | 0.03 |
| 11 | n/a | M182T; A224T | 1 | 0.03 |
| 12 | n/a | A224T | 1 | 0.03 |
| 14 | TEM-191 | E239K | 1 | 0.03 |

* n/a: not any

^1^: NCBI Bacterial Antimicrobial Resistance Reference Gene Database

**Supplementary Table 6.** Prevalence of plasmids in countries of provenance.

| **Country** | **GDP* (US $)** | **Number of isolates** | **% pConj with *tetM*** | **% markerless pConj** | **% p*bla*** |
| --- | --- | --- | --- | --- | --- |
| **LOW-INCOME ECONOMIES‡** | |  |  |  |  |
| The Gambia | 712 | 5 | 40 | 20 | 20 |
| Guinea | 885 | 1 | 100 | 0 | 100 |
| Guinea Bissau | 778 | 23 | 47.82608696 | 0 | 34.78261 |
| Tanzania | 1050 | 2 | 50 | 0 | 0 |
| Uganda | 643 | 1 | 100 | 0 | 0 |
| *TOTAL / AVERAGE %* | | *32* | *67.6* | *4* | *30.9* |
| **LOWER-MIDDLE INCOME ECONOMIES‡** | | |  |  |  |
| Angola | 3432 | 1 | 0 | 0 | 0 |
| Bhutan | 3360 | 7 | 42.85714286 | 0 | 14.28571 |
| Cape Verde | 3654 | 2 | 100 | 0 | 100 |
| India | 2015 | 22 | 9.090909091 | 9.0909091 | 22.72727 |
| Indonesia | 3893 | 4 | 75 | 25 | 75 |
| Kenya | 1710 | 103 | 66.01941748 | 0.9708738 | 53.39806 |
| Morocco | 3237 | 3 | 0 | 33.333333 | 0 |
| Pakistan | 1472 | 15 | 26.66666667 | 20 | 20 |
| Philippines | 3103 | 13 | 7.692307692 | 38.461538 | 46.15385 |
| Vietnam | 2564 | 6 | 50 | 16.666667 | 33.33333 |
| *TOTAL / AVERAGE %* | | *176* | *37.7* | *14.3* | *36.4* |
| **UPPER-MIDDLE-INCOME ECONOMIES‡** | | |  |  |  |
| Armenia | 4212 | 1 | 0 | 0 | 0 |
| Argentina | 11652 | 1 | 0 | 0 | 0 |
| Belarus | 6290 | 23 | 13.04347826 | 8.6956522 | 0 |
| Brazil | 8920 | 2 | 0 | 50 | 0 |
| Bulgaria | 9272 | 1 | 0 | 0 | 0 |
| China | 9771 | 8 | 12.5 | 25 | 12.5 |
| Cuba | 8541 | 4 | 50 | 25 | 0 |
| Ecuador | 6344 | 1 | 0 | 0 | 0 |
| Jamaica | 5355 | 2 | 50 | 50 | 0 |
| Malaysia | 11239 | 3 | 0 | 33.333333 | 66.66667 |
| Romania | 12301 | 1 | 0 | 100 | 100 |
| Russia | 11289 | 16 | 0 | 18.75 | 0 |
| South Africa | 6339 | 3 | 0 | 66.666667 | 33.33333 |
| Turkey | 9311 | 4 | 25 | 0 | 0 |
| Thailand | 7274 | 24 | 45.83333333 | 16.666667 | 41.66667 |
| *TOTAL / AVERAGE %* | | *94* | *13* | *14.3* | *16.9* |
| **HIGH-INCOME ECONOMIES‡** | |  |  |  |  |
| Australia | 57305 | 18 | 5.555555556 | 22.222222 | 11.11111 |
| Austria | 51512 | 1 | 0 | 0 | 0 |
| Canada | 46125 | 26 | 0 | 15.384615 | 3.846154 |
| Chile | 15923 | 2 | 0 | 0 | 0 |
| Denmark | 60596 | 8 | 0 | 25 | 0 |
| Estonia | 22928 | 18 | 16.66666667 | 0 | 27.77778 |
| Finland | 49960 | 1 | 0 | 0 | 0 |
| France | 41463 | 4 | 0 | 0 | 0 |
| Germany | 48196 | 6 | 16.66666667 | 16.666667 | 0 |
| Greece | 20324 | 5 | 20 | 60 | 20 |
| Hungary | 15938 | 1 | 0 | 100 | 0 |
| Ireland | 77449 | 2 | 0 | 0 | 50 |
| Italy | 34318 | 27 | 0 | 0 | 0 |
| Japan | 39287 | 23 | 0 | 0 | 0 |
| South Korea | 31362 | 1 | 0 | 0 | 0 |
| New Zealand | 41966 | 1 | 0 | 100 | 0 |
| Norway | 81807 | 14 | 28.57142857 | 14.285714 | 28.57143 |
| Poland | 15424 | 34 | 17.64705882 | 11.764706 | 5.882353 |
| Saudi Arabia | 23219 | 1 | 0 | 0 | 0 |
| Slovakia | 19546 | 1 | 0 | 100 | 0 |
| Slovenia | 26234 | 23 | 13.04347826 | 13.043478 | 13.04348 |
| Spain | 30524 | 89 | 0 | 2.247191 | 4.494382 |
| Sweden | 54112 | 9 | 11.11111111 | 0 | 0 |
| The Netherlands | 52978 | 5 | 20 | 0 | 20 |
| UK | 42491 | 2114 | 6.149479659 | 12.677389 | 8.751183 |
| USA | 62641 | 841 | 5.588585018 | 3.3293698 | 3.210464 |
| *TOTAL / AVERAGE %* | | *3275* | *6.1* | *19.1* | *7.5* |

* Gross Domestic Product (GDP) per capita per annum.

**‡** Country classification according to the World Bank for the 2020 fiscal year (https://datahelpdesk.worldbank.org/knowledgebase/articles/906519-world-bank-country-and-lending-groups)
